# Particularité de la cystinose infantile chez l'enfant tunisien

**DOI:** 10.11604/pamj.2015.22.348.7727

**Published:** 2015-12-11

**Authors:** Manel Jellouli, Hadhami Ben Turkia, Kamel Abidi, Yosra Hammi, Tahar Gargah

**Affiliations:** 1 Service de Pédiatrie, Hôpital Charles Nicolle, Tunis, Tunisie; 2 Service de Pédiatrie, Hôpital La Rabta, Tunis, Tunisie

**Keywords:** Enfant, insuffisance rénale, cystinose, cysteamine, syndrome de fanconi, infant, renal failure, cystinosis, cysteamine, Fanconi syndrome

## Abstract

La cystinose est une maladie rare qui résulte d'un défaut d'expression de la cystinosine transporteur de la cystine du lysosome. La forme infantile est la plus fréquente et la plus sévère. Elle conduit en dehors du traitement à l'insuffisance rénale chronique terminale au cours de la première décade de la vie. Nous rapportons l'expérience tunisienne de la cystinose infantile. Une étude rétrospective sur une période de 25 ans (1990-2014) était menée. Nous avons colligé 8 dossiers de cystinose infantile dans les services de pédiatrie des hôpitaux Charles Nicolle de Tunis et la Rabta de Tunis. Il s'agissait de 5garçons et de 3 filles. L’âge moyen au début des symptômes était de 6,37 mois (2-14 mois). L’âge moyen au moment du diagnostic était de 4 ans (7 mois-6 ans). Les dépôts cornéens de cystine étaient observés chez 7 patients. Sept patients présentaient une hypothyroïdie. La cystéamine était prescrite chez 6 patients. L’âge moyen au moment de la prescription de cystéamine était de 5,12 ans (8 mois- 13 ans). L’âge moyen lors de passage en insuffisance rénale chronique était de 3,4 ans. L’âge moyen lors du passage en insuffisance rénale chronique terminale était de 6,37 ans Actuellement, un patient garde une fonction rénale normale, trois patients sont en insuffisance rénale, deux patients sont décédés et un patient était transplanté. Il faut instaurer dans notre pays les moyens de diagnostic pour traiter tôt la maladie.

## Introduction

La cystinose est une maladie rare héréditaire à transmission autosomique récessive, qui résulte d'un défaut d'expression de la cystinosine transporteur de la cystine du lysosome vers le cytoplasme. Ceci conduit à une perturbation de fonctionnement cellulaire dans la plupart des organes et plus particulièrement le rein. Le gène en cause CTNS est situé sur le chromosome 17p13, comporte 12 exons et code pour la cystinosine [1, 2]. Plus que 60 mutations de ce gène ont été détectées. Trois formesde cystinose ont été décrites: la forme infantile, la plus fréquente et la plus sévère, la forme juvénile, moins sévère et d’évolution plus lente et la forme tardive ou adulte qui est sans retentissement rénal [3]. La cystinose infantile se manifeste dans la première année de la vie par un syndrome De Toni-Debré-Fanconi et progresse en dehors du traitement vers l'insuffisance rénale chronique terminale au cours de la première décade de la vie [4]. Le diagnostic de la cystinose s'appuie sur le dosage leucocytaire de la cystine permettant aussi la surveillance et l'ajustement thérapeutique. Le diagnostic est aussi possible par l’étude génétique [5]. L'introduction de la cystéamine dans les années 1980 a complètement changé le pronostic, permettant de retarder les complications rénales et extra-rénales mais cela n´en supprime pas certaines comme l´atteinte visuo-spatiale, la myopathie en particulier les troubles de la déglutition et possiblement l´atteinte neurologique centrale, l´hypogonadisme [6, 7]. A notre connaissance, aucune étude n'a concernée la cystinose infantile en Tunisie. Nous rapportons, dans le présent travail, l'expérience tunisienne de la cystinose infantile.

## Méthodes

Une étude rétrospective sur une période de 25 ans (1990-2014) était menée. Nous avons colligé 8 dossiers de cystinose infantile dans les services de pédiatrie des hôpitaux Charles Nicolle de Tunis et la Rabta de Tunis. Nous avons recueilli les données cliniques, biologiques et évolutives de la maladie. Le diagnostic de cystinose était retenu devant un dosage de cystine intra-leucocytaire > à 2 nmol d'hémicystine /mg de protéine chez 2 patients. Le dosage, non réalisable dans notre pays, était pratiqué au service de biochimie B, de l'hôpital Necker Enfants Malades, Paris, France. Le diagnostic, en dehors du dosage, était retenu devant la présence de syndrome De Toni Debré Fanconiet des dépôts cornéens de cystine chez 6 patients.

## Résultats

Il s'agissait de 5 garçons et de 3 filles. Les patients appartenaient à 7 familles. Dans une famille, il y avait une fille et son cousin atteints de cystinose. La consanguinité était notée dans 50% des cas. Un seul patient avait des antécédents de décès au bas âge dans la fratrie. L’âge moyen au début des symptômes était de 6,3 mois avec des extrêmes allant de 2 à 14mois. L’âge moyen au moment du diagnostic était de 3,6 ans avec des extrêmes allant de 7 mois à 6 ans. Les dépôts cornéens de cystine étaient observés chez 7 patients (Tableau 1). Tous les patients présentaient une tubulopathie proximale avec une polyurie, une fuite de l'eau et des électrolytes, une glucosurie et une protéinurie. Le rachitisme était présent dans tous les cas. Le retard de croissance était constaté chez tous les patients. Une kératopathie était notée chez une patiente. Sept patients présentaient une hypothyroïdie avec un âge moyen d'installation de cette endocrinopathie de 7,2 ans. Un patient âgé de 6 ans, sans antécédents néonataux particuliers, présentait une atrophie cortico-sous corticale avec hydrocéphalie à l'imagerie cérébrale demandée au cours d'exploration d'un retard psychomoteur. Une atteinte myogène était notée chez une patiente à l’âge de 6 ans. Tous les patients ont reçu un traitement symptomatique comportant des boissons abondantes, une supplémentation en potassium, en phosphore, en vitamine D et en hormones thyroïdiennes si hypothyroïdie. Une patiente était mise sous hormones de croissance. Cinq patients avaient reçu de l'indométacine. Aucun des patients n'a reçu de la cystéamine collyre. La première prescription de la cystéamine datait de 1999. La cystéamine était prescrite chez 6 patients. Les 2 autres patients étaient décédés au moment du diagnostic. L’âge moyen au moment de la prescription de cystéamine était de 5,8 ans (8 mois-13 ans) (Tableau 2). Deux patients ont eu un contrôle de la cystine intra ‘leucocytaire et un ajustement des doses a été effectué. Un diagnostic anténatal réalisé sur un échantillon de villosités choriales dosant la cystine leucocytaire était effectué au cours de deux grossesses montrant des fœtus sains.

**Tableau 1:** Caractéristiques des enfants ayant une cystinose

Patient n°	Sexe	Age au début des symptômes (mois)	Age au moment du diagnostic (année)	Age au moment de découverte de l'IRC (année)	Age au moment de passage en IRCT (année)	Examen ophtalmologique	Fonction thyroïdienne
1	Garçon	5	4	4	7	Dépôts au niveau de la cornée, la rétine	Hypothyroïdie
2	Garçon	6	5	5	5,5	Photophobie, dépôts cornéens	Hypothyroïdie
3	Garçon	5	4			Dépôts au niveau de la cornée, la rétine	Hypothyroïdie
4	Fille	6	5	5	6	dépôts cornéens	Hypothyroïdie
5	Garçon	6	6	5		Photophobie, dépôts cornéens	Hypothyroïdie
6	Fille	14	4	4	5	Dépôts cornéens	Hypothyroïdie
7	Fille	7	0,58	4		Kératopathie, dépôts cornéens	Hypothyroïdie
8	Garçon	2	0,66			Normal	Normale

IRC: insuffisance rénale chronique IRCT: insuffisance rénale chronique terminale

**Tableau 2:** Traitement par la cystéamine

Patient n°	Année de prescription	Age au moment de la prescription	Dose (mg/kg)	Cystine intra leucocytaire (nmol/mg protéine)	Taux de cystéamine(mmol/l)
1	1999	13 ans	48,3	++	++
2	1999	6 ans	33,3	++	++
3	+				
4	+				
5	2013	6 ans	44	++	++
6	2012	4 ans	43,2	++	++
7	2010	13 mois	44,7	2,9	1,1
8	2012	8 mois	49,3	2,1	++

+non traité; ++ non mesuré(e)

L’âge moyen lors de passage en insuffisance rénale chronique était de 4,5 ans. L’âge moyen lors du passage en insuffisance rénale chronique terminale était de 5,87 ans. Actuellement, un patient garde une fonction rénale normale, trois patients sonten insuffisance rénale, deux patients sont décédés et un patient était transplanté. Il s'agissait d'un garçon né en 1986. Le diagnostic de cystinose était fait en 1990 devant l'association d'anomalie tubulaire et la présence de dépôts cornéens de cystine. A l’âge de 4 ans, une insuffisance rénale chronique s'est installée et à l’âge de 7 ans. Il a eu une greffe rénale à partir d'un donneur cadavérique en 1999 soit à l’âge de 13 ans. Il a présenté une infection à CMV, 3 mois après la greffe et un diabète non insulino-dépendant. Une ponction biopsie du greffon a été effectuée devant une dégradation transitoire de la fonction rénale a objectivé de la fibrose sans signes de rejet. La prescription de la cystéamine fut en 1999, 5 mois après la transplantation rénale. Actuellement, il est âgé de 28 ans. L’âge du greffon est de 15 ans. La fonction rénale est stable avec un taux de la créatinine sanguine à 123 µmol/L.

## Discussion

La cystinose est une pathologie héréditaire rare. Dans notre étude, sur une période de 25 ans, nous n'avons isolé que 8 cas de cystinose infantile. Or, dans notre pays, le taux de mariage consanguin reste élevé. Cette pathologie serait sous diagnostiquée dans notre pays et ceci devant les difficultés à établir le diagnostic, puisque le dosage de la cystine intraleucocytaire et l’étude génétique de la cystinose ne s'effectuent pas dans notre pays. Les premiers symptômes de la cystinose infantile débutent précocement vers l’âge de 6 mois associant un syndrome de Toni-Debré-Fanconi avec un retard de croissance et une hypotrophie [8]. Dans notre série, l’âge moyen au début des symptômes était de 6,37 mois, mais 6 patients étaient diagnostiqués après l’âge de 4 ans.

D'autres organes peuvent être atteints au cours de la cystinose infantile. L'atteinte oculaire se manifeste vers l’âge de 2 à 4 ans à type de photophobie, dépôts de cystine dans la cornée mais également dans les autres structures de l’œil, des anomalies de la pigmentation de la rétine et une altération de l'acuité visuelle [9, 10]. Dans notre série, c'est l'association des anomalies tubulaires avec les dépôts cornéens de cystine qui a permis de retenir le diagnostic dans la majorité des cas.

Le traitement par la cystéamine a transformé le pronostic de cette maladie, qui en dehors du traitement aboutit à l'insuffisance rénale terminale au cours de la première décade de la vie [11, 12]. Gahl et al [13] ont montré que la cystéamine retardait l’évolution de l'insuffisance rénale et retardait voir limitait l'apparition des complications extra-rénales. Cependant, la prescription de la cystéamine doit être précoce d'où le diagnostic doit également être précoce. Sartorius et al [14] ont étudié 86 patients ayant une cystinose et ont montré clairement qu'un début de traitement par la cystéamine avant l’âge de 5 ans retardait l’évolution vers l'insuffisance rénale terminale. Thomas et al [15] ont étudié l’évolution de la cystinose dans les pays en voies de développement et les pays développés. Ils ont trouvé que dans les pays en voie de développement, la survie du rein natif était inférieure de 6,4 ans par rapport à celle des pays développés. Ceci a était expliqué par le défaut du traitement et le retard de la prescription de la cystéamine. Dans notre étude, nous avons observé une prescription tardive de la cystéamine avec une installation de l'insuffisance rénale terminale avant l’âge de 10 ans.

La transplantation rénale est indiquée lors du passage en insuffisance rénale terminale. L'indication de la néphrectomie des reins natifs dépend du degré de la polyurie [13, 16]. Des cristaux de cystine peuvent être observés sur le rein greffé [9, 10]. La survie du greffon chez les enfants ayant une cystinose est égale à celle observée chez les enfants transplantés pour autres pathologies [16]. Dans notre série, un seul patient était transplanté avec de bons résultats.

Les moyens de diagnostic de cystinose devraient être accessibles dans notre pays afin de diagnostiquer les patients atteints de cystinose précocement et ceci dans le but d'instaurer le traitement précocement et de retarder ainsi l’évolution vers l'insuffisance terminale. Le traitement est la greffe rénale, mais nous rencontrons également des difficultés devant un temps d'attente long dans notre pays.

## Conclusion

La cystinose est une pathologie rare dans notre pays. Certes sous diagnostiquée faute de moyens diagnostiques. Le traitement par cystéamine a complètement changé le pronostic de cette pathologie. Cependant, le pronostic dans notre pays demeure sévère devant un retard du diagnostic et des difficultés à procurer et à renouveler le traitement.
